# Revealing the Bioactive Potential of Romanian Wild Hop Cones: An Integrative Chemical, Antimicrobial, and Antibiofilm Activity and In Silico Docking Analysis

**DOI:** 10.3390/molecules31030405

**Published:** 2026-01-24

**Authors:** Mona Luciana Gălăţanu, Mariana Panţuroiu, Viorel Ordeanu, Răzvan Neagu, Roxana Măriuca Gavriloaia, Sorina Nicoleta Aurică, Gabriela Mariana Costache

**Affiliations:** 1 Faculty of Pharmacy, Titu Maiorescu University, Sincai Boulevard, No. 16, 040314 Bucharest, Romania; luciana.galatanu@prof.utm.ro (M.L.G.); sorina.aurica@gmail.com (S.N.A.);; 2 Regenerative Medicine Laboratory, “Cantacuzino” National Military Medical Institute for Research and Development, 103 Spl. Independentei, 050096 Bucharest, Romania

**Keywords:** *Humulus lupulus* (hop), essential oil, α-humulene, β-caryophyllene oxide, antioxidant, antimicrobial, biofilm, molecular docking, Sortase A

## Abstract

Hop (*Humulus lupulus* L.) is recognized as a valuable source of bioactive compounds; however, the phytochemical composition and biological potential of wild Romanian hops remain insufficiently characterized. In this study, the bioactive profile of wild hop cones was evaluated using an integrated phytochemical, biological, and in silico approach. The hydroethanolic extract was characterized by a total phenolic content of 25.61 mg GAE/g DW and a total flavonoid content of 3.20 mg RE/g DW, with α-acids predominating (8.77%) and β-acids detected only at trace levels (0.15%). Hydrodistillation yielded 0.613 ± 0.11% essential oil, which was rich in sesquiterpene hydrocarbons (64.61%), mainly α-humulene, β-caryophyllene oxide, selina-3,7-diene, and germacrene B. The hydroethanolic extract exhibited strong antioxidant activity (IC_50_ = 5.03 µg GAE/mL), whereas the essential oil showed a moderate but dose-dependent radical-scavenging capacity (IC_50_ = 0.44% *v*/*v*). In addition, the essential oil displayed pronounced antibacterial and antibiofilm activity against *Staphylococcus aureus*, *Escherichia coli*, and *Pseudomonas aeruginosa*, at 25 mg/mL, with the highest antibiofilm inhibition observed for *Pseudomonas aeruginosa* (96.44%). Molecular docking analysis suggested that the major volatile constituents may interact with *Staphylococcus aureus* Sortase A, providing a plausible mechanistic basis for the observed antibiofilm effects. Overall, these findings indicate that wild Romanian hop cones represent a promising source of antioxidant and antimicrobial bioactive compounds, supporting their potential applications in pharmaceutical, food, and cosmetic formulations, as well as in natural-product-based drug discovery.

## 1. Introduction

*Humulus lupulus* L. (hop) is a climbing species native to Romania, predominantly in humid, shaded areas such as riparian forests, riverbanks, and alluvial meadows, where ecological conditions support its vigorous growth [1]. Hop is a deciduous, flowering plant belonging to the Cannabaceae family. The leaves are simple, opposite or alternate, with three or five lobes, acuminate tips, and serrate margins. The male flowers appear in June and have no petals. The female flowers appear in late August or September and are grouped in inflorescences called strobiles, which are cone-like and consist of membranous stipules and bracts attached to a hairy axis. Each small branch of the axis bears a bract, represented only by its pair of stipules, which subtend either four or six bracts, each enclosing a flower. The fruit is an achene that does not split at maturity [2].

The plant is widely used in the brewing industry and in phytotherapy due to its diverse pharmacological potential. Female cones of hop contain a rich array of bioactive compounds, including alpha bitter acids (humulone and its derivatives), beta bitter acids (lupulone and its derivatives), prenylated chalcones (xanthohumol, isoxanthohumol), polyphenols, and essential oil [3,4]. These constituents have been reported to exhibit antibacterial, antiviral, and antifungal effects, contributing to the protective role of hop extracts against microbial contamination These effects were verified through in vitro studies on cultures of Gram-positive bacteria, including strains of *Staphylococcus*, as well as the methicillin-susceptible one and on resistant strains as *Micrococcus, Streptomycetes, Corynebacterium*, *Enterococcus,* and *Mycobacterium* [5,6]. Beyond their traditional sedative and phytoestrogenic uses, hop-derived compounds have attracted increasing attention for their antimicrobial and antivirulence potential [7,8,9].

Although the phytochemical composition of cultivated European hop varieties has been extensively investigated, considerably less attention has been paid to wild hop populations growing under non-domesticated conditions. Wild hops are exposed to distinct environmental pressures, including variable climate, soil composition, and biotic stress, which may influence the accumulation and relative distribution of bioactive compounds. As a result, wild hop cones may exhibit quantitative and qualitative differences in bitter acids, polyphenols, and volatile constituents compared with cultivated genotypes selected primarily for brewing performance.

Exploring the phytochemical profile of Romanian wild hop populations therefore offers an opportunity to identify alternative chemotypes and bioactive patterns that are not fully represented in cultivated European hops. This aspect is particularly relevant in the context of biofilm-associated infections, which remain difficult to treat using conventional antimicrobial therapies. Biofilms are structured microbial communities embedded in a self-produced extracellular polymeric substance (EPS) matrix composed of proteins, polysaccharides, and extracellular DNA. This matrix promotes microbial adhesion and confers increased tolerance to host immune responses and antimicrobial agents by limiting phagocytosis and drug penetration. In *Staphylococcus aureus*, biofilm initiation and surface colonization are critically dependent on Sortase A (SrtA), a membrane-anchored transpeptidase responsible for the covalent attachment of LPXTG-containing surface proteins to the peptidoglycan layer. These surface proteins are essential for bacterial adhesion, host interaction, and early biofilm development [10,11,12]. Consequently, targeting SrtA has emerged as a promising antivirulence strategy that interferes with biofilm formation without exerting strong bactericidal pressure.

Natural hydrophobic compounds, particularly terpenoids, have attracted increasing attention as potential antivirulence agents due to their ability to interact with bacterial membranes and surface-associated enzymes. Hop essential oil (*Humulus lupulus* L.) is characterized by a high content of sesquiterpenes, such as α-humulene and β-caryophyllene oxide, which possess physicochemical properties favorable for penetrating biofilm matrices and interacting with hydrophobic protein pockets. On this basis, hop essential oil was selected for antibiofilm evaluation in the present study, and SrtA was chosen as a biologically relevant molecular target to explore a plausible mechanistic link between essential oil composition and the observed antibiofilm effects.

The aim of this study was to investigate the phytochemical profile and biological potential of *Humulus lupulus* L. by quantifying total polyphenols, flavonoids, and α-/β-acids, as well as by isolating and characterizing the essential oil obtained by hydrodistillation using GC–MS analysis. The major constituents identified support the biological findings, as the hydroethanolic extract and essential oil exhibited in vitro antioxidant activity, while the essential oil also showed antimicrobial and antibiofilm effects. In addition, molecular docking analyses of the major volatile constituents of the essential oil (α-humulene, β-caryophyllene oxide, selina-3,7-diene, and germacrene B) were performed against *Staphylococcus aureus* Sortase A to provide a mechanistic perspective on the experimentally observed antibiofilm activity. By integrating chemical characterization, biological evaluation, and in silico analysis, this study provides a comprehensive assessment of wild hop cones as a source of bioactive compounds with potential applications in the food, pharmaceutical, and cosmetic sectors.

## 2. Results

### 2.1. Phytochemical Assays

The results of the polyphenol, flavone, and α- and β-acid quantitative determinations are presented in Table 1.

The phytochemical analysis of hop cones revealed a total flavonoid content (TFC) of 3.20 mg RE/g DW and a total phenolic content (TPC) of 25.61 mg GAE/g DW. These values confirm that hop cones are a rich source of polyphenolic compounds, with flavonoids representing a substantial fraction of the total phenolics.

A pronounced imbalance was observed between the two classes of bitter acids, with α-acids occurring in high abundance (8.77%), while β-acids were detected only in low concentrations (0.15%). This distribution reflects the characteristic chemical profile of hop cones and suggests that α-acids may contribute more substantially to the species’ biological properties.

### 2.2. Hydrodistillation and GC-MS Analysis of the Essential Oil

By hydrodistillation, a light-yellowish essential oil with a specific, dense, and bitter smell was obtained. The essential oil yield was 0.613 ± 0.11% (mL/100 g dried mass of hop cones).

The classes of bioactive compounds of the hop cones’ essential oil revealed through GC-MS analysis were sesquiterpene hydrocarbons (64.61%), while oxygenated sesquiterpenes represented 18.03%, as presented in Table 2 and Figure S1 from the Supplementary Material. Aliphatic oxygenated compounds (aldehydes, ketones, esters) account for 10.3%, and only 3.05% constitute monoterpene hydrocarbons, while aromatics/other hydrocarbons (polyene, azulene, indene) represent 3.99% of the total composition.

**Figure 1:**
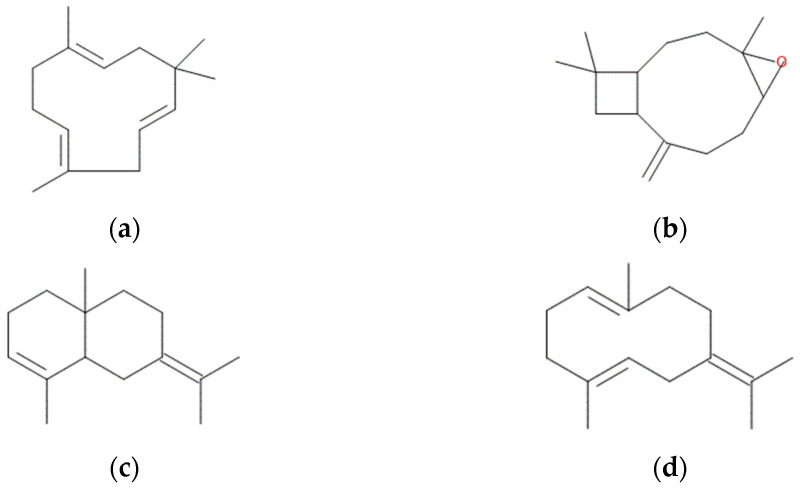
The main bioactive compounds from the hop cones essential oil. (**a**) α-humulene; (**b**) β-caryophyllene oxide; (**c**) selina-3,7-diene; (**d**) germacrene B.

The predominant constituents of the hop cone essential oil were α-humulene (41.68%), followed by β-caryophyllene oxide (14.80%), selina-3,7-diene (5.16%), and germacrene B (4.76%), as illustrated in Figure 1, which highlights the main bioactive compounds identified in the essential oil.

### 2.3. Biological Activity Determinations

#### 2.3.1. Antioxidant Capacity

**Figure 2:**
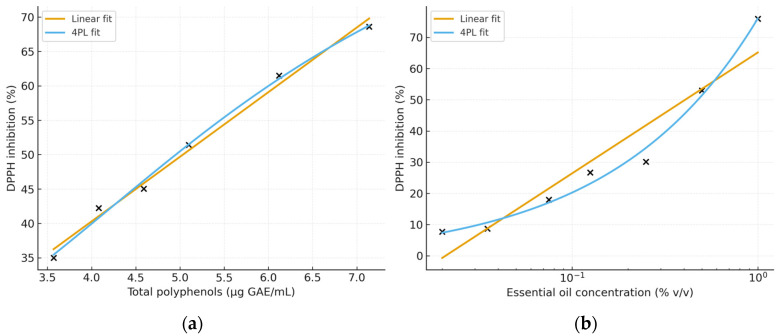
DPPH radical scavenging activity of hop cones with linear and nonlinear (4PL) IC_50_ curve fitting. (**a**) hop cone ethanolic extract; (**b**) hop cone essential oil.

Linear regression analysis of the hydroethanolic hop cone extract revealed a strong concentration–response relationship (R^2^ = 0.97) and yielded an IC_50_ of 5.03 µg GAE/mL, while nonlinear fitting using a four-parameter logistic (4PL) model provided a comparable IC_50_ value of 5.70 µg GAE/mL. The fitted inhibition curves obtained with both approaches are shown in Figure 2, demonstrating a consistent dose-dependent antioxidant response. Overall, the extract exhibited clear antioxidant activity across the tested concentration range, indicating efficient DPPH radical scavenging attributable to its polyphenolic components.

The hop cone essential oil exhibited a clear concentration-dependent DPPH radical scavenging effect, with inhibition values ranging from 75.93% at 1% (*v*/*v*) to 7.73% at 0.020% (*v*/*v*) (Figure 2). The dose–response curve showed a typical sigmoidal decrease across the tested concentration range, with the steepest decline in activity observed between 0.25% and 0.50% (*v*/*v*), indicating that this interval brackets the 50% inhibition threshold. Based on the experimental inhibition values, the IC_50_ was initially estimated by linear interpolation between the two concentration points flanking the 50% inhibition threshold, yielding an IC_50_ of 0.47% (*v*/*v*). To obtain a more robust, model-based estimate, the data were further fitted with a four-parameter logistic (4PL) regression, yielding an IC_50_ of 0.44% (*v*/*v*). The close agreement between the linear and nonlinear estimates confirms the consistency of the experimental data and the reliability of the dose–response relationship.

Overall, the hop essential oil demonstrated moderate antioxidant activity, consistent with its chemical composition, which is dominated by mono- and sesquiterpenes, which typically exhibit moderate radical-scavenging efficiency compared with polyphenol-rich plant extracts. The obtained IC_50_ values indicate that although the essential oil is not a strong DPPH scavenger, it contributes meaningfully to the antioxidant profile of hop-derived products.

#### 2.3.2. Antimicrobial and Antibiofilm Activity

**Figure 3:**
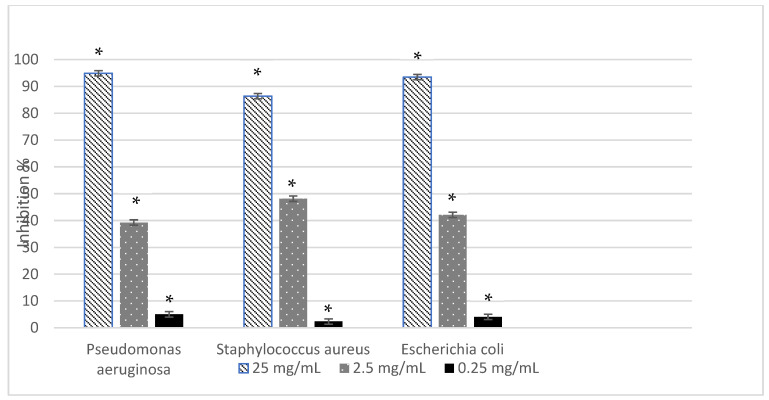
Antibacterial activity of hop cone essential oil. Values and are expressed as mean ± SD (*n* = 3). Statistically significant differences compared to the solvent control (*p* < 0.05).

**Figure 4:**
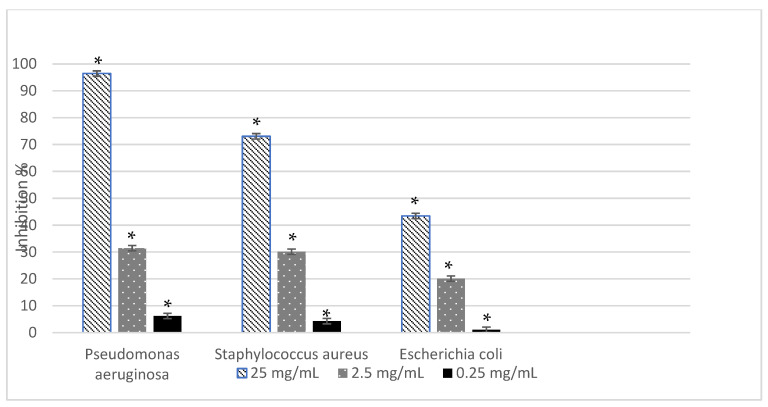
Antibiofilm activity of hop cone essential oil. Values are expressed as mean ± SD (*n* = 3). Statistically significant differences compared to the solvent control (*p* < 0.05).

The inhibitory efficacy of hop cone essential oil against bacterial growth and biofilm formation was calculated relative to the untreated control, which was considered as 100% growth or biofilm formation. Maximum inhibitory effects were observed at the highest tested concentration (25 mg/mL), as shown in Figure 3 and Figure 4.

At this concentration, the essential oil exhibited strong antibacterial activity against all tested strains, with inhibition values ranging from 86.36% for *Staphylococcus aureus* to 94.91% for *Pseudomonas aeruginosa*, while *Escherichia coli* showed an intermediate response (93.49%). The antibiofilm assay revealed a strain-dependent response. The highest inhibition of biofilm formation was observed for *P. aeruginosa* (96.44%), followed by *S. aureus* (73.08%) and *E. coli* (43.43%), indicating a reduced antibiofilm susceptibility of *E. coli* compared to the other strains.

#### 2.3.3. Molecular Docking of Hop Essential Oil Constituents with Sortase A

**Figure 5:**
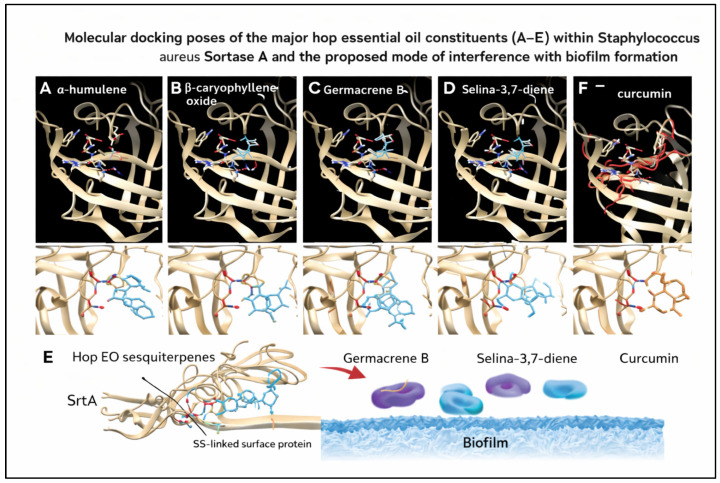
Molecular docking poses of the major hop essential oil constituents—(**A**) α-humulene, (**B**) β-caryophyllene oxide, (**C**) germacrene B, (**D**) selina-3,7-diene—and the reference compound curcumin (**F**) within the active site of *Staphylococcus aureus* Sortase A. Docked ligands are shown within the catalytic pocket adjacent to the Cys184–His120–Arg197 triad.

Molecular docking simulations were performed for the major volatile constituents of hop essential oil—α-humulene, β-caryophyllene oxide, germacrene B, and selina-3,7-diene—against *Staphylococcus aureus* Sortase A (SrtA), using curcumin as a reference inhibitor (Figure 5). For each ligand, multiple binding modes were generated, and the lowest-energy pose was selected for comparison. Curcumin displayed the highest binding affinity toward SrtA, with a minimum docking score of −7.5 kcal/mol, confirming its suitability as a positive control and supporting the reliability of the docking protocol. The best-ranked poses of curcumin showed consistent clustering, indicating satisfactory convergence of the docking simulations.

Among the hop-derived compounds, α-humulene exhibited the most favorable binding affinity (−6.9 kcal/mol), followed by germacrene B (−6.6 kcal/mol) and β-caryophyllene oxide (−6.5 kcal/mol), while selina-3,7-diene showed slightly weaker binding (−6.2 kcal/mol), resulting in the following affinity ranking: curcumin > α-humulene > germacrene B ≈ β-caryophyllene oxide > selina-3,7-diene. All hop sesquiterpenes were accommodated within the catalytic pocket or adjacent hydrophobic groove of SrtA. Visualization in UCSF Chimera revealed that α-humulene penetrates deeply into the hydrophobic tunnel adjacent to the Cys184–His120–Arg197 catalytic triad, where it is stabilized predominantly by van der Waals interactions. β-Caryophyllene oxide adopted a comparable binding orientation, with the presence of the epoxide moiety enabling a more directed positioning within the active site. The binding energies obtained fall within the range commonly reported for natural SrtA inhibitors and indicate moderate but consistent interactions with the enzyme.

Overall, these docking results support the experimental observations of antibiofilm activity for hop essential oil and suggest that its biological effects may arise from the combined contribution of multiple volatile constituents interacting with Sortase A.

## 3. Discussion

The results of this study provide insights into the phytochemical profile and biological activities of hop cones, enabling a comparative discussion with previously reported data.

In the present study, hop cone extracts contained 25.61 mg GAE/g DW of total phenolic compounds and 3.20 mg RE/g DW of flavonoids. These values are consistent with those reported by Kowalska et al., who found up to 4.20% total phenolics and approximately 0.37% flavonoids in hop cones [13]. The total phenolic content determined in the analyzed extracts exceeded the values previously reported for methanolic extracts (7.12 mg GAE/g) and was higher than the range of 11.9–21.2 mg/g described for field-grown hop cones, while remaining lower than the concentrations achieved using optimized hydroethanolic extraction protocols (approximately 44 mg GAE/g).

In contrast, the total flavonoid content measured in the present samples (3.20 mg RE/g DW) was lower than the values commonly reported for hop cone extracts, which typically range between 10 and 20 mg RE or QE/g. Such discrepancies may be attributed to differences in hop genotype, cultivation, and environmental conditions, as well as to methodological factors, including solvent polarity, extraction protocol, and the reference standards used for quantification (rutin equivalents versus quercetin equivalents) [14,15,16].

Bitter acids, particularly α- and β-acids, are recognized for their significant contribution to the pharmacological potential of hop-derived products. A pronounced imbalance was observed between the two classes of bitter acids in the Romanian wild hop, with α-acids occurring in high abundance while β-acids were detected only in low concentrations. Compared with the ranges reported for cultivated hop varieties (α-acids 2–18% and β-acids 1–10%), our sample showed an α-acid level (8%) well within the expected interval, whereas the β-acid content (0.15%) was markedly lower than typical values described in the literature [17,18,19]. Although the β-acid concentration was markedly lower than values typically reported for cultivated hop varieties, the overall bitter-acid profile may still be regarded as adequate. This is particularly relevant, given that the analyzed sample originates from a wild hop population grown under non-optimized environmental and agronomic conditions. Moreover, the relatively high α-acid content observed in this Romanian wild hop genotype is noteworthy, as α-acids have been increasingly associated with beneficial neurocognitive [20,21,22], antimicrobial [23], protective [24,25], and anticancer [26] effects, according to recent clinical investigations. This enhances the functional relevance and the pharmacological potential of the Romanian wild hop sample, despite its non-domesticated origin.

In the present study, the essential oil yield from hop cones was determined to be 0.613 ± 0.11% (mL/100 g dry weight), a value close to those reported in the literature, although the percentage may vary significantly depending on the cultivar, growing conditions, and extraction method. For example, a study conducted in Italy reported an average yield of 0.82 ± 0.13% (mL/100 g dry weight), which is similar to the value obtained in our study [27]. Another study states that ‘Cluster’ hop cultivars have an average essential oil yield of 0.7–1.1 mL/100 g, whereas ‘Bullion’ cultivars can reach up to 2.5 mL/100 g [28].

In addition to α-acids, the volatile fraction of hop cones represents another key contributor to their biological activity. The essential oil of hop cones is composed of a complex mixture of terpenes and related compounds, encompassing monoterpene and sesquiterpene hydrocarbons as well as their oxygenated derivatives. The relative abundance and chemical diversity of these constituents significantly influence both the biological activities and the aromatic profile of the essential oil. This chemical composition differs from that of the hop cones from Italy, where α-humulene is also predominant (37.01%), but β-myrcene (26.85%), β-caryophyllene (13.74%), 2-undecanone (13.63%), and α-selinene (8.70%) are the following main components [27]. Similarly, the composition of essential oil is influenced by various factors, such as geographical region, climate, and soil characteristics. To avoid any overinterpretation, the present results indicate a largely comparable chemical composition and biological activity between Romanian wild hops and cultivated hops. The observed similarities suggest that, with respect to their major constituents, Romanian wild hops and cultivated hop varieties exhibit similar phytochemical and functional profiles. Potential differences related to population origin and ecological factors are likely subtle and may not be fully captured by GC–MS profiling or bulk biological screening. Therefore, further studies using advanced analytical approaches, such as metabolomic profiling and multivariate analysis, are required to clarify this variability.

Among these diverse constituents, α-humulene stands out for its abundance and its significant biological and aromatic properties. Few studies have shown antibacterial properties of α-humulene against strains of *Bacteroides fragilis, Staphylococcus aureus,* and *Escherichia coli* [29,30]. Becker and Holtmann (2024) have highlighted the anti-inflammatory action of α-humulene in a recent in vitro study, with a possible mechanism of reducing pro-inflammatory cytokines (interleukin-6) in lipopolysaccharide-induced THP-1 cells [31]. In another experimental study on mice and rats, Fernandes et al. observed that alpha-humulene diminished edema formation induced by histamine injection, demonstrating marked inhibitory effects [32]. It is important to highlight that α-humulene has been demonstrated to have significant antitumor activity against various cancer cell lines, including hepatocellular, colorectal, and breast cancers, by inducing apoptosis through the inhibition of Akt signaling pathways, inhibiting proliferation, and acting synergistically with other bioactive compounds [33,34]. Beyond its pharmacological actions, α-humulene contributes to the aromatic profile of the essential oil through earthy, woody, and spicy notes, which are highly relevant in brewing and fragrance applications [35]. β-Caryophyllene oxide, the second predominant compound in hop cones’ essential oil, an oxygenated sesquiterpene, has been reported to exhibit notable anti-inflammatory and anticancer activities by inducing apoptosis and ferritinophagy and modulating oxidative stress pathways [36,37]. Germacrene B, an abundant sesquiterpene hydrocarbon in hop cones, exhibits antioxidant and antimicrobial activities and plays an important role in plant defense mechanisms [38].

Beyond the contribution of individual volatile constituents, the antioxidant activity of hop cones was further evaluated at the extract level to capture the combined effect of phenolic compounds present in the polar fraction. The hydroethanolic hop cone extract exhibited a relevant antioxidant capacity, as indicated by the IC_50_ values obtained through linear (5.03 µg GAE/mL) and nonlinear (5.70 µg GAE/mL) modeling. These results are aligned with previous reports showing that the antioxidant activity of *Humulus lupulus* arises primarily from its polyphenolic constituents, including flavonoids, phenolic acids, and prenylated phenols. The close agreement between IC_50_ values derived from two independent approaches further supports the robustness of the assessment. Comparative data reported by Abram et al. indicated IC_50_ values of 0.005–0.010 mg/mL for hop cone extracts and approximately 0.020 mg/mL for hop leaves, suggesting that antioxidant capacity may vary with extraction method, plant maturity, genotype, and environmental conditions [39]. Compared with previously reported antioxidant activities of hop cone extracts—such as IC_50_ values of 0.12 mg/mL for the Saaz 3 cultivar or 0.079–0.139 mmol Trolox/g dw for Cascade and Columbus [40]—the IC_50_ values determined in this study, expressed per µg GAE/mL, reflect a high phenolic efficiency. Although direct quantitative comparisons are constrained by methodological differences, the strong radical-scavenging activity observed here indicates that wild Romanian hop cones possess an antioxidant capacity comparable to, or exceeding, that of several commercially cultivated hop varieties. In line with previous reports describing elevated FRAP, DPPH responses in polar hop extracts, the hydroethanolic cone extract exhibited pronounced antioxidant activity, emphasizing the importance of solvent polarity for the efficient recovery of phenolic constituents [41]. When expressed per µg GAE/mL, the IC_50_ values obtained in the present study reflect a high antioxidant efficiency of the phenolic fraction, indicating that relatively low amounts of phenolic compounds are sufficient to achieve substantial radical-scavenging activity. This enhanced efficiency may be associated with the specific phytochemical profile of Romanian wild hop cones and/or the effectiveness of the hydroethanolic extraction protocol employed.

The antioxidant evaluation of hop cone essential oil revealed a moderate, yet clearly dose-dependent, radical-scavenging effect. The IC_50_ values obtained by linear interpolation (0.47% *v*/*v*) and nonlinear four-parameter logistic (4PL) modeling (0.44% *v*/*v*) were in close agreement, supporting the robustness of the experimental data and indicating a well-defined concentration–response relationship. The moderate antioxidant activity of the essential oil is consistent with its sesquiterpene-dominated composition. Terpenes such as α-humulene, β-caryophyllene oxide, germacrene B, and selina-3,7-diene, although biologically active, generally exhibit lower hydrogen-donating capacity than polyphenolic antioxidants. Accordingly, essential oils from *Humulus lupulus* and other aromatic plants typically exhibit higher IC_50_ values in DPPH assays than polyphenol-rich extracts, reflecting the modest-to-moderate radical-scavenging activity characteristic of terpene-rich oils, particularly when complex mixtures of mono- and sesquiterpenes are involved [42,43,44]. Nevertheless, the IC_50_ value obtained indicates a meaningful ability of the hop essential oil to scavenge DPPH radicals, suggesting that volatile constituents may contribute additively or synergistically to the overall antioxidant profile of hop-derived products.

In addition to this antioxidant contribution, the volatile fraction is also highly relevant from an antimicrobial perspective, as the essential oil obtained from wild hop cones exhibited a chemical profile dominated by sesquiterpenes, particularly α-humulene and β-caryophyllene oxide, both of which are widely recognized for their antimicrobial potential. Consistent with this composition, the essential oil showed a strong and concentration-dependent antibacterial activity, with inhibition values exceeding 85% for all tested strains at 25 mg/mL. Among the investigated bacteria, *Pseudomonas aeruginosa* and *Escherichia coli* displayed slightly higher sensitivity than *Staphylococcus aureus* at elevated concentrations, suggesting differences in membrane permeability and cellular targets. Such mechanisms are especially relevant for Gram-positive bacteria like *Staphylococcus aureus*, whose thicker but more accessible peptidoglycan layer facilitates the penetration of terpenoids, although the notable susceptibility of Gram-negative strains indicates that the essential oil components may also effectively disrupt outer membrane structures.

Importantly, the essential oil demonstrated good to very good antibiofilm efficacy, indicating a capacity not only to inhibit bacterial growth but also to interfere with biofilm formation. This effect was clearly strain-dependent, with *P. aeruginosa* exhibiting the highest biofilm inhibition, followed by *S. aureus*, whereas *E. coli* showed a markedly reduced antibiofilm susceptibility. Moreover, the antibiofilm activity was strongly dose-dependent, suggesting the existence of a concentration threshold required to effectively interfere with biofilm development. Biofilm-associated resistance is largely driven by the extracellular polymeric substance (EPS) matrix, which protects bacteria from antibiotics and host immune responses. Therefore, the ability of hop essential oil to reduce biofilm formation highlights its potential relevance for controlling persistent infections. In this context, it should be emphasized that the biological activities reported in the present study refer to the essential oil as a chemically defined mixture; consequently, direct attribution of the observed effects to individual constituents requires future experimental validation using purified compounds.

To gain insight into potential molecular mechanisms underlying the observed antibiofilm activity, molecular docking was employed as a hypothesis-generating approach. The results indicate that the major volatile constituents—α-humulene, β-caryophyllene oxide, germacrene B, and selina-3,7-diene—can bind within the catalytic groove of *Staphylococcus aureus* Srt A, with predicted binding energies ranging from −6.9 to −6.2 kcal/mol. These values fall within the range reported for natural SrtA inhibitors and are consistent with moderate inhibitory potential [45,46,47].

The docking behavior of hop sesquiterpenes agrees with previous studies showing that hydrophobic plant-derived compounds preferentially interact with a compact hydrophobic groove [48,49,50] adjacent to the Cys184–His120–Arg197 catalytic triad through non-covalent interactions [51,52]. Among the compounds evaluated, α-humulene displayed the most favorable affinity, occupying the hydrophobic tunnel near the catalytic triad, a positioning consistent with steric blockage of the sorting-signal binding channel involved in surface protein anchoring and early biofilm development [52,53,54]. β-Caryophyllene oxide showed a comparable affinity, likely supported by additional stabilizing interactions enabled by its epoxide group.

The weaker affinities observed for selina-3,7-diene may reflect their less compact conformations and predominantly hydrocarbon character; however, their concurrent presence in the essential oil may contribute additively to the antibiofilm effects observed in vitro. Overall, these findings support the hypothesis that hop essential oil may exert antibiofilm activity, at least in part, through multi-ligand interference with Sortase A. It should be emphasized that molecular docking represents a hypothesis-generating approach requiring experimental validation; nevertheless, it provides a plausible molecular framework linking enzyme interaction with the observed antibiofilm effects. Such an antivirulence mode of action, targeting biofilm formation rather than bacterial viability, may complement conventional antibiotics and reduce selective pressure for resistance, thereby supporting the potential of hop-derived compounds as natural antimicrobial adjuvants [55].

Although the investigated compounds exhibited promising biological activities, several limitations warrant consideration. Antioxidant, antimicrobial, and antibiofilm activities were evaluated in relation to solvents, without direct comparison to established positive controls (e.g., ascorbic acid or curcumin), which should be included in future studies. Compound identification relied on GC–MS library matching, without further structural confirmation by LC–MS or authentic reference standards. Moreover, the essential oil was tested as a complex mixture, and it cannot be excluded that the observed biological effects may be driven predominantly by one or a limited number of major constituents rather than by true combined or synergistic interactions. Therefore, potential synergistic or antagonistic relationships among individual components remain to be clarified through fractionation and single-compound testing. Finally, molecular docking was employed as a hypothesis-generating approach and requires experimental validation. Addressing these limitations will further strengthen the mechanistic understanding and translational relevance of bioactive compounds derived from hops.

## 4. Materials and Methods

### 4.1. Materials

**Figure 6:**
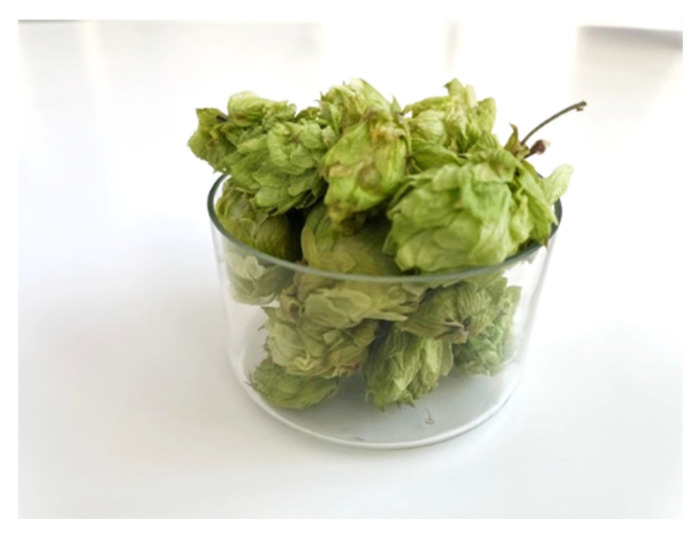
Female cones of *Humulus lupulus* L.

Female cones of wild hop (*Humulus lupulus* L.) were collected from hills around Breaza city, Prahova County, in August–September 2025. The species was taxonomically authenticated at the Botany Department, Titu Maiorescu University, Faculty of Pharmacy, Bucharest, Romania (Figure 6).

Strains of *Staphylococcus aureus* ATCC 25923, *Escherichia coli* ATCC 25922, and *Pseudomonas aeruginosa* ATCC 27853 were procured from “Cantacuzino” National Military Medical Institute for Research and Development, Bucharest, Romania.

### 4.2. Chemicals

All solvents and reagents were of analytical grade. Methanol, sodium hydroxide, Folin–Ciocalteu reagent, crystal violet, gallic acid, and rutin were obtained from Sigma-Aldrich (St. Louis, MO, USA). 2,2-Diphenyl-1-picrylhydrazyl (DPPH), Tween 20, and ethanol were purchased from Merck (Darmstadt, Germany). Culture media Mueller–Hinton agar was supplied by Thermo Fisher Scientific (Dreieich, Germany).

### 4.3. Preparation of the Extract

For total phenol and flavonoid assays and antioxidant activity, an ethanolic extract was made. Dried and ground hop cones (1 g) were extracted with 100 mL of 50% (*w*/*w*) ethanol under reflux in an electric water bath at 100 °C for 30 min. The mixture was filtered through an ashless filter paper, adjusted to a final volume of 100 mL in a volumetric flask, and stored at 5 °C until further analysis.

### 4.4. Phytochemical Assays

#### 4.4.1. Total Phenolic Content

The total polyphenolic content of the hop cone extracts was determined spectrophotometrically using the Folin–Ciocalteu method, with gallic acid as the calibration standard [56,57]. Briefly, 1 mL of extract was combined with 4.5 mL of deionized water, 2.5 mL of diluted Folin–Ciocalteu reagent, and 2 mL of a 7% sodium carbonate solution. After a 30-min incubation at room temperature, the absorbance was recorded at 765 nm using a VWR UV-6300 PC spectrophotometer (VWR International, Vienna, Austria). The total polyphenolic content was calculated based on a gallic acid calibration curve (with R^2^ = 0.999728) and expressed as milligrams of gallic acid equivalents per gram of dry weight (mg GAE/g DW). All assays were performed in triplicate.

#### 4.4.2. Total Flavonoid Content

The total flavonoid content of the extracts was determined spectrophotometrically using the aluminum chloride method in the presence of sodium acetate [58,59]. 10 mL of ethanolic extract was diluted with methanol and filtered. An aliquot of 5 mL of diluted extract was mixed with 5 mL of sodium acetate solution (100 g/L) and 3 mL of aluminum chloride solution (25 g/L). The volume was adjusted to 25 mL with methanol, homogenized, and incubated at room temperature for 15 min. Absorbance was measured at 430 nm using a VWR UV-6300 PC spectrophotometer. Flavonoid content was calculated from a rutin calibration curve (having R^2^ = 0.99952) and expressed as milligrams of rutin equivalents per gram of dry weight (mg RE/g DW). All measurements were performed in triplicate.

#### 4.4.3. Alpha and Beta Acids Content

The quantification of total α- and β-acids was performed through separate extraction from the hop samples, followed by spectrophotometric analysis at three distinct wavelengths [17,18].

A 2.5 g sample of finely ground hops was extracted with 50 mL of methanol by magnetic stirring for 30 min at room temperature, followed by a 10 min resting period. The mixture was then filtered through a 0.45 μm Millipore membrane. A 50 μL aliquot of the filtrate was diluted to 25 mL with methanolic NaOH (0.5 mL of 6 M NaOH in 250 mL methanol). The resulting solution was analyzed in a quartz cuvette (1 cm path length), with a blank prepared by adding 50 μL of methanol to 25 mL of methanolic NaOH. Absorbance was measured at 275, 325, and 355 nm, using a VWR UV-6300 PC spectrophotometer. The bitter acid content was obtained with the following equations:

(1) $$\alpha -acid\;content\left(\%\right)=\left(-51.26\cdot A355\;nm\right)+\left(73.79\cdot A325\;nm\right)-(19.07\cdot A275\;nm)$$

(2) $$\beta -acid\;content\left(\%\right)=\left(55.27\cdot A355\;nm\right)-\left(47.59\cdot A325\;nm\right)+(5.1\cdot A275\;nm)$$

where A = absorbance reading at each wavelength.

All analyses were carried out in triplicate.

### 4.5. Hydrodistillation and GC-MS Analysis of the Essential Oil

Fresh, finely ground hop cones underwent hydro-distillation in a closed system using a glass Clevenger-type apparatus for 3 h. For each distillation, 150 g of plant material was mixed with 600 mL of distilled water, according to the procedure described in the 10th edition of the European Pharmacopoeia [60], which ensures accurate volumetric assessment of the essential oil. The experiment was performed in triplicate.

To determine the exact yield of the essential oil, the moisture content and loss-on-drying of the raw plant material were assessed. Hop cones were placed in a desiccator over anhydrous sodium sulfate (R) at atmospheric pressure and room temperature until a constant weight was achieved. The moisture content was calculated as the difference between the mass before and after drying. The essential oil content was expressed as a percentage relative to the dried hop cones using the following formula:

(3) $$yield\;\left(content\;of\;essential\;oil\right)=\frac{volume\;of\;essential\;oil\;\left(mL\right)}{mass\;of\;dryhop\;cones\;\left(g\right)}\;\times \;100$$

The main constituents of the essential oil were identified using Gas Chromatography–Mass Spectrometry (GC–MS). Analyses were carried out on a Thermo Electron Corporation Focus gas chromatograph equipped with a splitter and coupled to a Thermo Electron Corporation DSQII mass spectrometer (Thermo Scientific, Waltham, MA, USA). Separation was achieved on a Macrogol 20,000 capillary column (30 m length, 0.25 mm internal diameter, 0.25 μm film thickness). Helium was used as the carrier gas at a flow rate of 1.5 mL/min, and the injection volume was maintained at 1.0 μL. The column oven temperature was planned to rise gradually from 65 °C to 200 °C throughout the 60 min analytical run. Quantification was based on the integration of chromatographic peak areas, while identification was achieved by comparing the obtained mass spectra with reference spectra from the Wiley 8 and NIST 07 libraries [61,62].

### 4.6. Biological Activity Determinations

#### 4.6.1. Antioxidant Activity (DPPH Radical Scavenging Assay)

The antioxidant activity of both the hydroethanolic extract and the essential oil obtained from hop cone hops was evaluated using the 2,2-diphenyl-1-picrylhydrazyl (DPPH) radical scavenging assay, a widely used method for assessing antioxidant capacity. The assay is based on the reduction of the stable DPPH• radical, which results in a color change from deep violet to yellow and a corresponding decrease in absorbance at 517 nm [63,64].

- Hydroethanolic Hop Extract

The antioxidant activity of the hydroethanolic hop cone extract was evaluated using the DPPH radical scavenging assay. A stock solution of the extract, previously standardized to its total phenolic content, was used throughout the experiment. Aliquots of the extract stock solution (0.35–0.70 mL), corresponding to final concentrations of 3.5–7.1 µg GAE/mL in the reaction mixture, were transferred into 25 mL volumetric flasks. Each flask received 3 mL of DPPH solution (0.1 mM in methanol), and the volume was adjusted to 25 mL with methanol. The mixtures were incubated for 30 min in the dark at room temperature, after which absorbance was measured at 517 nm using a VWR UV-6300 PC UV–Vis spectrophotometer. Methanol was used as the blank, while the control consisted of the DPPH solution prepared under identical conditions without extract. The percentage of DPPH radical scavenging was calculated relative to the control. The IC_50_ value was determined by plotting the inhibition percentages against the final concentration of total phenolics (µg GAE/mL) present in the reaction mixture and applying linear regression analysis.

- Hop essential oil

The antioxidant activity of hop essential oil was assessed using a method adapted for hydrophobic samples. A stock solution of 1% (*v*/*v*) essential oil was prepared by dissolving 1 mL of essential oil in methanol and adjusting the final volume to 100 mL. Serial dilutions were prepared to obtain final concentrations of 1.00, 0.50, 0.25, 0.125, 0.075, 0.035, and 0.020% (*v*/*v*). A fresh 0.1 mM DPPH solution in methanol was adjusted to an initial absorbance of 0.90 ± 0.05 at 517 nm. For each assay, 2.0 mL of the DPPH solution was mixed with 1.0 mL of the essential oil dilution in a cuvette (1 cm path length). Blanks were prepared by replacing the DPPH solution with methanol. Samples were incubated for 30 min in the dark at room temperature before absorbance was measured at 517 nm.

The percentage of DPPH inhibition was calculated using Equation (4):

(4) $$\begin{array}{cccc} & \mathrm{DPPH}\;\mathrm{inhibition}\;(\%)=\frac{A_{\mathrm{control}}-A_{\mathrm{sample}}}{A_{\mathrm{control}}}\times 100 & & \end{array}$$

where

$A_{\mathrm{control}}\;$ is the absorbance of the DPPH solution without sample, and
$A_{\mathrm{sample}\;}$ is the absorbance of the reaction mixture containing extract or essential oil.

The antioxidant activity was expressed as IC_50_, defined as the concentration required to inhibit 50% of the DPPH radical. IC_50_ values were determined using linear interpolation between the two experimental data points bracketing 50% inhibition, and nonlinear regression using a four-parameter logistic (4PL) model fitted to % inhibition versus the logarithm of sample concentration. The 4PL model, widely recognized as a robust method for dose–response analysis, was used as the reference for reporting IC_50_ values. All measurements were performed in triplicate, and data are expressed as mean ± standard deviation.

#### 4.6.2. Antimicrobial and Antibiofilm Activity

The antimicrobial activity of the hop essential oil was assessed using the microdilution method in sterile 96-well flat-bottom microplates containing Mueller–Hinton broth. The bacterial inoculum was prepared according to the Clinical and Laboratory Standards Institute (CLSI) guidelines, using the direct colony suspension method [65]. Briefly, 24 h agar-grown colonies were resuspended in 0.9% saline and adjusted to a 0.5 McFarland standard, corresponding to approximately 10^8^ CFU/mL [66].

The essential oil was incorporated into a 30% (*w*/*w*) oil-in-water (O/W) emulsion using Tween 20 (0.5%) as an emulsifier. The emulsion was diluted in sterile double-distilled water to obtain a stock solution of 25 mg/mL, followed by serial decimal dilutions. After 24 h incubation at 37 °C, bacterial growth was quantified by measuring the absorbance at 562 nm using an EnSight Multimode Plate Reader (PerkinElmer, Waltham, MA, USA). Antimicrobial efficacy was calculated using the equation:

(5) $$\mathrm{Efficacy}\;(\%)=100-(\frac{\mathrm{Sample}\;\mathrm{absorbance}}{\mathrm{Reference}\;\mathrm{absorbance}})\times 100$$

The reference absorbance was calculated as:

(6) $$\mathrm{Reference}=A_{\mathrm{positive}\;\mathrm{control}}+A_{\mathrm{negative}\;\mathrm{control}}-A_{\mathrm{blank}}$$

where

Positive control = bacteria + medium, without essential oil,
Negative control = medium without bacteria,
Blank = solvent without bacteria.

The efficacy was evaluated as very good (≥90%), good (75–89%), moderate (50–74%), satisfactory (25–49%), and unsatisfactory (0–24%).

Biofilm inhibition and biofilm disruption were evaluated using the crystal violet (CV) staining method. After incubation with the essential oil dilutions, wells were washed twice with sterile distilled water and stained with 0.1% CV for 15 min at room temperature. Excess dye was removed, and plates were air-dried, followed by drying at 50 °C for 60 min. Bound CV was solubilized, and absorbance was measured at 570 nm using the EnSight Multimode Plate Reader. Lower absorbance values indicate reduced biofilm biomass. The efficacy was considered very good (≥90%), good (75–89%), moderate (50–74%), satisfactory (25–49%), and unsatisfactory (0–24%).

The antibacterial and antibiofilm inhibition values presented correspond to the maximum inhibitory effect observed across the tested concentration range, which occurred at 25 µg/mL for all assays. The obtained values represent the mean ± SD of three independent experiments (n = 3), and differences compared to the solvent control (sterile double-distilled water) were considered statistically significant at *p* < 0.05.

#### 4.6.3. Molecular Docking of Hop Essential Oil Constituents with Sortase A

Molecular docking analysis was performed using AutoDock Vina (version 1.2.3). The crystal structure of *Staphylococcus aureus* Srt A (PDB ID: 1T2W) was retrieved from the RCSB Protein Data Bank and prepared using AutoDockTools 1.5.7 by removing crystallographic water molecules, adding polar hydrogens, and assigning Kollman united-atom charges and appropriate AutoDock atom types [67,68,69]. The essential oil constituents (α-humulene, β-caryophyllene oxide, germacrene B, and selina-3,7-diene) were energy-minimized, assigned Gasteiger partial charges, and converted to PDBQT format with automatically defined rotatable bonds. The docking grid was centered at (−20.874, −13.933, −19.323) Å with dimensions of 24.468 × 24.018 × 26.334 Å^3^, defined to fully encompass the Srt A catalytic pocket and adjacent binding groove. Docking calculations were performed with an exhaustiveness value of 64 and a maximum of 100 binding modes generated per ligand to ensure adequate conformational sampling and convergence of docking solutions. The lowest-energy binding pose for each ligand was selected for further analysis.

To validate the docking protocol, a positive-control docking was performed using curcumin, a reported natural inhibitor of Srt A [52,70]. Curcumin was docked under the same grid parameters and docking settings as the hop-derived ligands. The control ligand reproduced binding within the Srt A catalytic groove, supporting the reliability and reproducibility of the docking workflow.

Protein–ligand interactions and binding orientations were visualized using UCSF Chimera (version 1.19).

### 4.7. Statistical Data Processing

All experimental data were expressed as mean ± standard deviation (SD) and obtained from three independent experiments (n = 3). Statistical analysis was performed using XLSTAT software (version 2022.4.5). Differences among groups were evaluated by one-way analysis of variance (ANOVA), followed by Dunnett’s post hoc test for comparison with the solvent control Differences were considered statistically significant at *p* < 0.05.

## 5. Conclusions

This study provides an integrated phytochemical, biological, and in silico evaluation of Romanian wild hop cones, highlighting their potential as a source of bioactive compounds with antioxidant and antimicrobial relevance. The hydroethanolic extract showed strong radical scavenging activity, consistent with its high polyphenol content, while the essential oil, rich in sesquiterpenes such as α-humulene, germacrene and β-caryophyllene oxide, exhibited pronounced antibacterial and antibiofilm activity against *Pseudomonas aeruginosa*, followed by *Staphylococcus aureus* and *Escherichia coli.* The essential oil also displayed a moderate, dose-dependent antioxidant effect, in line with its terpene-dominated composition. Molecular docking, applied as a hypothesis-generating approach, suggested that the major sesquiterpenes can interact with the catalytic pocket of *Staphylococcus aureus* Sortase A, providing a plausible mechanistic framework for the observed antibiofilm effects. Further studies are warranted to experimentally validate the proposed molecular mechanisms.

Although the phytochemical profile of wild hop cones is broadly comparable to that of cultivated hops, these findings highlight wild populations as an underexplored source of bioactive natural products with potential applications in the pharmaceutical, food, and cosmetic fields.

## Figures and Tables

**Table 1:** Total phenolic content (TPC), total flavonoid content (TFC) α-, and β-acid content in hop cones.

TPC (mg GAE/g DW)	TFC (mg RE/g DW)	α-Acid Content %	β-Acid Content %
25.61 ± 0.02	3.20 ± 0.05	8.77± 0.007	0.15 ± 0.003

**Table 2:** GC-MS analysis of the essential oil of hop cones.

No.	Compound	Retention Time (min)	Area (min)	Area [%]
1	Myrcene	11.245	1,152,299.825	3.05
2	Nonaldehyde	21.819	875,454.647	2.32
3	2-Methyl-hexadecanal	27.094	205,137.635	0.54
4	β-Caryophyllene oxide	28.053	5,399,268.552	14.80
5	Azulene	28.346	521,386.068	1.38
6	2-Undecanone	28.570	1,133,910.205	3.00
7	4-Methyl-decenoate	29.410	512,483.171	1.36
8	α-Humulene	30.216	15,731,415.243	41.68
9	Muurolene	30.883	536,717.160	1.42
10	β-Patchoulene	31.039	430,720.443	1.14
11	Eudesma-4,7(14)-11-diene	31.614	1,405,668.726	3.72
12	γ-Gurjunene	31.791	1,329,138.104	3.52
13	Cadina-1,4-diene	32.852	1,209,856.356	3.21
14	Selina-3,7-diene	33.298	1,947,271.467	5.16
15	2-Tridecanone	34.526	488,920.421	1.30
16	Germacrene B	34.614	1,796,923.960	4.76
17	3-Cyclohexen-carboxaldehyde	39.896	479,370.854	1.27
18	Globulol	40.920	191,539.36	0.51
19	Nonadecatriene	41.192	727,835.740	1.93
20	1-H-Indene	41.896	257,795.907	0.68
21	Methyl 4,7,10,13-hexadecatetraenoate	42.749	191,654.129	0.51
22	α-Eudesmol	44.276	469,586.395	1.24
23	β-Eudesmol	44.447	400,628.367	1.06
24	Neointermedeol	44.960	158,055.457	0.42
Total:			37,739,095.263	100

## Data Availability

The original contributions presented in this study are included in the article/Supplementary Material. Further inquiries can be directed to the corresponding authors.

## Supplementary Materials

The following supporting information can be downloaded at: https://www.mdpi.com/article/10.3390/molecules31030405/s1, Figure S1: GC-MS chromatogram of hop cones essential oil.
