# Supplementary figures and images for: Revealing the Bioactive Potential of Romanian Wild Hop Cones: An Integrative Chemical, Antimicrobial, and Antibiofilm Activity and In Silico Docking Analysis
Source: Molecules. 2026 Jan 24;31(3):405. doi: 10.3390/molecules31030405 (PMC12898592; doi:10.3390/molecules31030405)

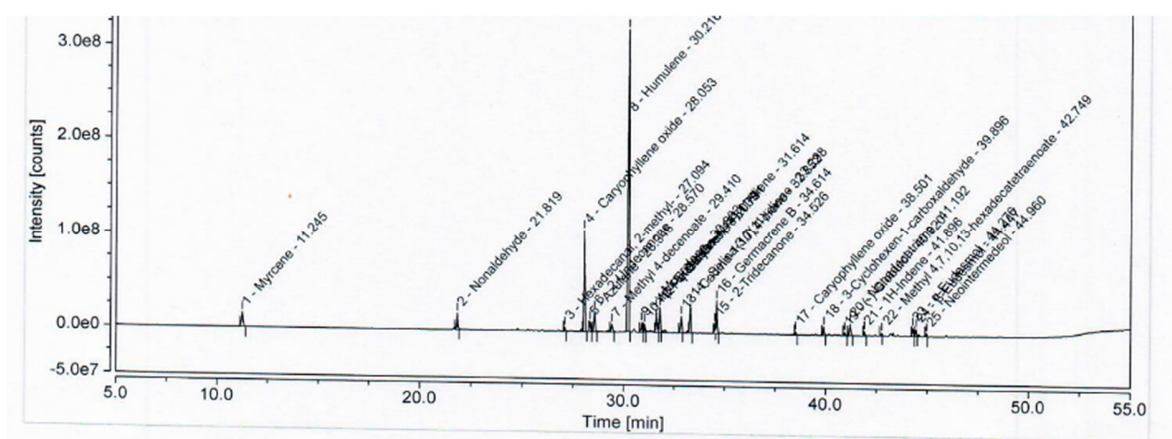

**Figure S1.** GC-MS chromatogram of hop cones essential oil

Supplement: Supplementary file 1 [file molecules-31-00405-s001.zip › molecules-4074698-supplementary.pdf]
